# Rapid Bone Healing after Intentional Replantation of a Molar with Apical Actinomycosis 

**DOI:** 10.22037/iej.v13i1.19369

**Published:** 2018

**Authors:** Saeed Asgary, Leyla Roghanizadeh

**Affiliations:** a *Iranian Center For Endodontic Research, Research Institute of Dental Sciences, Dental School, Shahid Beheshti University of Medical Sciences, Tehran, Iran*

**Keywords:** Actinomycosis, Calcium-enriched Mixture, CEM Cement, Endodontic, Tooth Replantation, Periapical Periodontitis

## Abstract

Actinomycosis is a rare lesion of the jaws and may present as periapical pathosis; therefore, it is essential to be correctly diagnosed and managed. This case presentation describes management of a tooth with a symptomatic apical periodontitis caused by *Actinomyces* species supplemented with medicine prescription. A woman was referred for endodontic management of tooth #19. The tooth had a history of previous nonsurgical endodontic retreatment. Clinically, the tooth was very sensitive to percussion. Radiographic evaluation showed a large periapical lesion. Intentional replantation (IR) was planned. The tooth was atraumatically extracted. Without any curettage, through the blood flow coming out of the socket, a small yellowish granule was detected and sent for examination. After root-end preparations, the cavities were filled with calcium-enriched mixture cement and the tooth was carefully replanted. Histopathological assessment proved actinomycosis sulfur granule. According to infectious disease specialist recommendation, low-dose and long-term penicillin V was prescribed. Interestingly, at 2-month follow-up, remarkable bone healing was observed. In the cases of apical actinomycosis, IR in combination with antibiotic therapy, even without the curettage of the lesion, may be successfully employed.

## Introduction

Actinomycosis is an uncommon chronic infectious disease. The disease caused by species of *Actinomyces*, which are gram-positive, slow growing, opportunistic, anaerobic or microaerophilic filamentous organisms [1]. *Actinomyces* may live in the human oropharynx, gastrointestinal tract and urogenital tract as normal flora. Between the species, *Actinomyces (A.) israelii* is the most prevalent pathogen*. *The bacteria establishes a granulomatous inflammation distinguishable by swelling, sinus tract, and purulent discharge containing yellowish sulfur granule [2].

A rupture in the mucosal barrier, or presence of necrotic tissues, can facilitate entry and spread of the infection [3]; cervicofacial region is the most involved site. Also thoracic, abdominal, and pelvic regions, and even central nervous and musculoskeletal systems can be infected [1]. In oral and cervicofacial actinomycosis, histories of trauma, poor oral hygiene, tooth extraction, invasive dental operations or bisphosphonate related osteonecrosis of the jaws may be present [2-6]. Predisposing factors are debilitating conditions such as malignancy, radiation therapy, diabetes, malnutrition and immunosuppression [2, 5].

**Figure 1:**
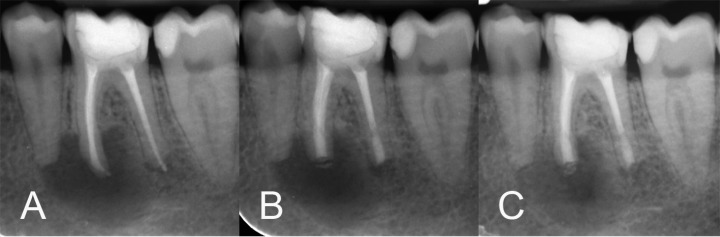
*A)* Diagnostic preoperative radiography of the mandibular left first molar exhibits a large radiolucency around the mesial root and a small one around the distal root; *B)* Immediate postoperative radiograph after retrograde treatment and intentional replantation; *C) *2-month follow-up radiograph showing remarkable bone healing in the periapical region

The role of actinomycotic organisms in failure of root canal treatment has been documented [3, 7-11]. Their involvement in remission and exacerbation of symptoms is highly suspicious [3]. They may cause re-emerging of symptoms and sinus tracts after standard endodontic treatment. Species of this organism can live and endure in periapical tissues as extraradicular infection leading to perpetuate the periapical lesions [12]. 

Intentional replantation (IR) technique defines as extraction of an endodontically involved tooth, doing treatment procedures extra orally, and insertion of the tooth back into its alveolar socket. When endodontic surgery is necessary, but is not possible or practical, IR is a viable treatment option; with careful case selection, it has high success rate [13]. 

This manuscript is report of a patient who suffered from an endodontically retreated mandibular molar with a large apical lesion. Treatment plan was decided to be retrograde procedure *via* IR of the tooth.

## Case Report

A 22-year-old woman was referred with the complaint of having trouble with her mandibular left first molar for a while. Because of unsuccessful root canal treatment (RCT), non-surgical retreatment had done for the tooth by an endodontist. However, even after the retreatment, discomfort remained. The medical history was non-contributory. There was no history of soft tissue swelling, abscess formation or sinus tract. The teeth had a composite resin restoration. In clinical examination, normal mucosa in the adjacent vestibule could be seen. The tooth was tender so to percussion. The probing depth was normal, with no mobility. Radiographic evaluation (Figure 1A) exhibited previous root canal therapy with a good quality. It also revealed a large radiolucency around the mesial root and a small one in the apex of distal root. The clinical diagnosis was symptomatic apical periodontitis in a previously retreated tooth. The treatment options including tooth extraction and implant replacement, root-end surgery or IR were described to the patient. She decided to go under IR and preserve the tooth. An informed consent was obtained.

Treatment procedure was performed by an endodontist. On the day of operation, after a 0.12% chlorhexidine mouth rinse, local mandibular block anaesthesia with 2% lidocaine plus 1:80000 epinephrine (Darupakhsh, Tehran, Iran) administered. Then, the tooth with a slow rocking motion was extracted with a dental forceps. A 2×2 mm creamy yellowish-white granule was washed out from the socket through blood. It was immersed in 10% formalin solution and was sent for oral pathologist. 

Holding the tooth by the crown using the forceps, ~2 mm root apices were resected with a diamond bur in a high speed handpiece. Using Gates Glidden burs #2-4, 3-mm depth root-end cavities were prepared. The powder and liquid of the calcium-enriched mixture (CEM) cement were mixed according to manufacturer’s instructions and the mixture was inserted into the prepared cavities. After saline rinsing and aspiration of blood clot with suction, without any curettage, the tooth was carefully replanted into alveolar socket with the forceps. Normal occlusion was checked and precise repositioning confirmed with periapical radiography (Figure 1B). No splinting and occlusal adjustment was performed. The total extraoral operation time was <7 min. Postoperative instructions including careful oral hygiene, chlorhexidine mouth rinse and soft diet were given. 

**Figure 2:**
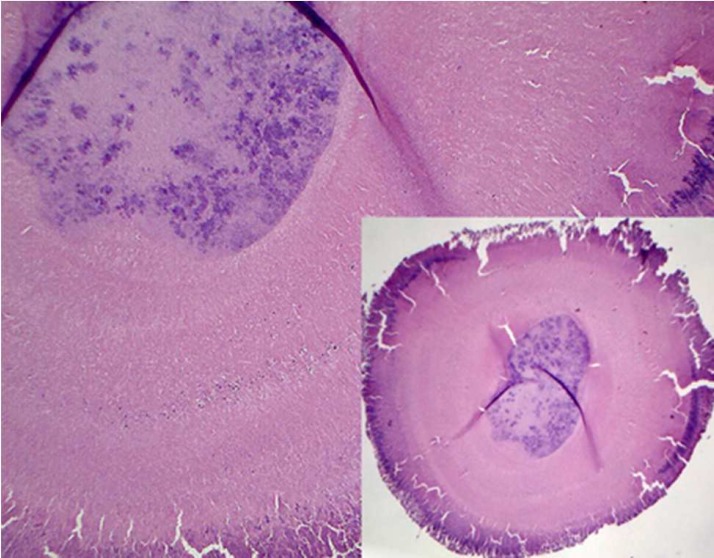
Bacterial aggregates manifest as structure containing gram-positive branching filamentous

The histopathological examination revealed sulfur granule specimen contained colonies of gram-positive branching filamentous organisms manifesting actinomycosis (Figure 2). A consultation was inquired from an infectious disease specialist; a long-term and low-dose administration of oral penicillin V 500 mg/day for 2 months was prescribed. The post-operation clinical recovery was acquired thoroughly and the patient was symptom-free. In radiographic evaluation at the 2-month follow-up, remarkable bone healing was observed (Figure 1C).

## Discussion

Periapical actinomycosis is not a prevalent periradicular infection. Although the infection mostly involve cervicofacial region [14], only a few cases of periapical actinomycosis have been published [3, 8, 10, 11, 15]. It has been believed that this infection would be treated successfully if the involved tooth is extracted [7, 11], or by excision of the periapical lesion *via* an endodontic flap surgery [7-9, 11] combined with antibiotic therapy [3]. In our case, however, due to performing IR, the periradicular lesion was not exposed/curetted and consequently, excisional biopsy was not obtained. 

Reaching the right diagnosis to this infection is a burdensome task [6]. In histopathological examinations, sulfur granules, manifesting as aggregations of filamentous gram-positive organisms, highly supports differential diagnosis of actinomycosis [1, 2, 14]. Undoubted diagnosis is obtained by isolating the bacteria despite high failure rate of culture [1, 2, 6, 14] or by 16s rRNA sequencing of the organisms [2, 14, 16] from a tissue specimen, pus or a sulfur granule. The majority of apical actinomycosis cases have been diagnosed on the basis of the presence of sulfur granules obtained through apical surgery or tooth extraction, except some doing microbiologic cultures [7, 15]. In this case the diagnosis was based on identification of the sulfur granule in histopathological examination. 

One of the characteristics of actinomycosis infections is forming sinus tracts with purulent discharges [5]. These indicating signs have been reported in cases of periapical actinomycosis too [7, 8, 10, 11]. On the contrary, our case presents a periapical actinomycosis without any sinus tract and/or purulent discharge. 

A long-term study about healing of periapical lesions demonstrated that the *Actinomyces* species were present in the failed cases and in the most of failures, no other organism was proved to contribute [17]. Periapical actinomycosis, in the category of extraradicular infections can contribute to persistence of apical lesions even after high quality endodontic (re)treatments [18] ,which can be a continuum or independent of the intraradicular infections [11]. The extraradicular infections are not accessible by conventional orthograde procedures. The source of infection in the cases of apical actinomycosis might be intraradicular biofilm or extraradicular bacterial aggregations such as the sulfur granule. In this case, it seems that the source of infection was the sulfur granule; therefore, previous non-surgical endodontic retreatment had not able to eliminate it.

In spite of any surgical debridement and curettage, this case demonstrated favorable treatment outcomes and considerable bone healing after just two months was obtained. This can be considered as a fast bone healing process which shows natural healing power of human bodies in the absence of etiologic factors. Healing after an endodontic surgery needs regeneration of periodontium, medullary/cortical bone regeneration and new cementum formation [19]. An investigation, inspected osseous response to removal of periradicular and radicular tissues from the alveolus of rhesus monkeys. At 14 days, most of the excisional defects were filled with newly formed woven bone trabeculae, and the trabeculae were more mature with a functioning periosteum in regeneration of the cortical plate at just 28 days post-operation [20]. 

Long-term drug therapy is required for curing actinomycosis infections [6]. Cervicofacial actinomycosis are usually treated by debridement of necrotic tissues and antibiotic therapy [1, 7, 9, 11]. In contrast with actinomycosis in other sites of the body, some articles mentioned no need for antibiotic therapy in apical actinomycosis after endodontic surgery or tooth extraction [8, 11]. In the present case, instantly after the surgical treatment no antibiotic was given; however, according to an infectious disease specialist, low-dose/long-term penicillin V prescribed which resulted in satisfactory healing of the pathology. Penicillin with different dosage/treatment duration has still been considered the treatment of choice for this infection [1, 2, 6]. There are also reports of periapical actinomycosis with successful treatment outcomes that amoxicillin was prescribed [3, 8, 11].

Biocompatibility and sealing ability are necessary qualities for root-end filling materials in modern endodontics. CEM has demonstrated favourable characteristics in terms of sealability, cementogenesis/osteogenesis and maturogenesis [21-25]; these features can promote healing of radicular/periradicular tissues.

## Conclusion

Apical actinomycosis should be in mind as one of the reasons of persistent apical periodontitis. Even when curettage of the defect could not be carried out, IR might be considered as a successful treatment plan for such involved teeth. Antibiotic therapy could help elimination of the infection.

## References

[1] Moghimi M, Salentijn E, Debets-Ossenkop Y, Karagozoglu KH, Forouzanfar T (2013). Treatment of cervicofacial actinomycosis: a report of 19 cases and review of literature. Med Oral Patol Oral Cir Bucal.

[2] Valour F, Sénéchal A, Dupieux C, Karsenty J, Lustig S, Breton P, Gleizal A, Boussel L, Laurent F, Braun E (2014). Actinomycosis: etiology, clinical features, diagnosis, treatment, and management. Infect Drug Resist.

[3] Tseng S-K, Tsai Y-L, Li U-M, Jeng J-H (2009). Radicular cyst with actinomycotic infection in an upper anterior tooth. J Formos Med Assoc.

[4] Barnard D, Davies J, Figdor D (1996). Susceptibility of Actinomyces israelii to antibiotics, sodium hypochlorite and calcium hydroxide. Int Endod J.

[5] Panda R, Kumar V, Saha S, Choudhary L, Pandey A (2017). Unusual Behavior of a Posttraumatic Scar: Craniofacial Actinomycosis. Wounds.

[6] Volante M, Contucci A, Fantoni M, Ricci R, Galli J (2005). Cervicofacial actinomycosis: still a difficult differential diagnosis. Acta Otorhinolaryngol Ital.

[7] Sundqvist G, Reuterving C-O (1980). Isolation of Actinomyces israelii from periapical lesion. J Endod.

[8] Al-Hezaimi K (2010). Apical actinomycosis: case report. J Can Dent Assoc.

[9] Jamshidi D, Moazami F, Sobhnamayan F, Taheri A (2015). Clinical and Histopathologic Investigation of Periapical Actinomycosis with Cutaneous Lesion: a Case Report. J Dent.

[10] Pasupathy SP, Chakravarthy D, Chanmougananda S, Nair PP (2012). Periapical actinomycosis. BMJ case reports.

[11] Ricucci D, Siqueira JF (2008). Apical actinomycosis as a continuum of intraradicular and extraradicular infection: case report and critical review on its involvement with treatment failure. J Endod.

[12] Nair P (2006). On the causes of persistent apical periodontitis: a review. Int Endod J.

[13] Asgary S, Alim Marvasti L, Kolahdouzan A (2014). Indications and case series of intentional replantation of teeth. Iran Endod J.

[14] Wong V, Turmezei T, Weston V (2011). Actinomycosis. BMJ.

[15] O'Grady JF, Reade PC (1988). Periapical actinomycosis involving Actinomyces israelii. J Endod.

[16] Siqueira J, Rôças I, Moraes S, Santos K (2002). Direct amplification of rRNA gene sequences for identification of selected oral pathogens in root canal infections. Int Endod J.

[17] Sjögren U, Figdor D, Persson S, Sundqvist G (1997). Influence of infection at the time of root filling on the outcome of endodontic treatment of teeth with apical periodontitis. Int Endod J.

[18] Sjögren U, Happonen R, Kahnberg K, Sundqvist G (1988). Survival of Arachnia propionica in periapical tissue. Int Endod J.

[19] Apaydin ES, Shabahang S, Torabinejad M (2004). Hard-tissue healing after application of fresh or set MTA as root-end-filling material. J Endod.

[20] Harrison JW, Jurosky KA (1992). Wound healing in the tissues of the periodontium following periradicular surgery III The osseous excisional wound. J Endod.

[21] Hasheminia M, Loriaei Nejad S, Asgary S (2010). Sealing Ability of MTA and CEM Cement as Root-End Fillings of Human Teeth in Dry, Saliva or Blood-Contaminated Conditions. Iran Endod J.

[22] Yavari HR, Samiei M, Shahi S, Aghazadeh M, Jafari F, Abdolrahimi M, Asgary S (2012). Microleakage comparison of four dental materials as intra-orifice barriers in endodontically treated teeth. Iran Endod J.

[23] Samiee M, Eghbal MJ, Parirokh M, Abbas FM, Asgary S (2010). Repair of furcal perforation using a new endodontic cement. Clin Oral Investig.

[24] Rahimi S, Mokhtari H, Shahi S, Kazemi A, Asgary S, Eghbal MJ, Mesgariabbasi M, Mohajeri D (2012). Osseous reaction to implantation of two endodontic cements: Mineral trioxide aggregate (MTA) and calcium enriched mixture (CEM). Med Oral Patol Oral Cir Bucal.

[25] Nosrat A, Asgary S (2010). Apexogenesis of a symptomatic molar with calcium enriched mixture. Int Endod J.

